# The Impact of the Burden of COVID-19 Regulatory Reporting in a Small Independent Hospital and a Large Network Hospital: Comparative Mixed Methods Study

**DOI:** 10.2196/63681

**Published:** 2025-03-26

**Authors:** Yalini Senathirajah, David R Kaufman, Kenrick Cato, Pia Daniel, Patricia Roblin, Andre Kushniruk, Elizabeth M Borycki, Emanuel Feld, Poli Debi

**Affiliations:** 1 School of Medicine University of Pittsburgh Pittsburgh, PA United States; 2 Emergency Preparedness Division SUNY Downstate Health Sciences University Brooklyn, NY United States; 3 Children's Hospital of Philadelphia Philadelphia, PA United States; 4 Health Information Science University of Victoria Victoria, BC Canada

**Keywords:** regulatory reporting, human factors, reporting burden, emergency response, COVID-19, hospital resilience, pandemic response

## Abstract

**Background:**

During the COVID-19 pandemic in 2020, hospitals encountered numerous challenges that compounded their difficulties. Some of these challenges directly impacted patient care, such as the need to expand capacities, adjust services, and use new knowledge to save lives in an ever-evolving situation. In addition, hospitals faced regulatory challenges.

**Objective:**

This paper presents the findings of a qualitative study that aimed to compare the effects of reporting requirements on a small independent hospital and a large network hospital during the COVID-19 pandemic.

**Methods:**

We used both quantitative and qualitative analyses and conducted 51 interviews, which were thematically analyzed. We quantified the changes in regulatory reporting requirements during the first 14 months of the pandemic.

**Results:**

Reporting requirements placed a substantial time burden on key clinical personnel at the small independent hospital, consequently reducing the time available for patient care. Conversely, the large network hospital had dedicated nonclinical staff responsible for reporting duties, and their robust health information system facilitated this work.

**Conclusions:**

The discrepancy in health IT capabilities suggests that there may be significant institutional inequities affecting smaller hospitals’ ability to respond to a pandemic and adequately support public health efforts. Electronic certification guidelines are essential to addressing the substantial equity issues. We discuss in detail the health care policy implications of these findings.

## Introduction

### Background

This research compared the impact of reporting requirements on a small independent hospital (SIH) that served a medically underserved population during the COVID-19 pandemic with a large network hospital that served the same city. The SIH, which had 198 staffed beds, was designated as a COVID-19–only hospital by the State of New York from March 28, 2020, to June 5, 2020. A large network hospital (LNH) with 11 campuses and greater resources was used as a point of comparison. Both institutions are in New York City (NYC), and their experiences during the same period were compared. This research has important implications for government and regulatory bodies, highlighting the need to adjust policies to meet public health information needs without further straining an already overwhelmed health care system.

A core part of the mission of every public health agency is to consistently and methodically gather, compile, interpret, and disseminate data concerning the health status of the community [1]. Hospitals play a crucial role in supporting this mission by gathering and reporting data on their patient populations and institutional resources, which becomes particularly essential in public health crises. Approximately 96% of US hospitals have adopted certified electronic health record (EHR) systems, many with automated reporting for functions like public health surveillance [2]. This shift marks a major advance in the digitization of hospital records and the efficiency of data reporting, though challenges in interoperability still impede smooth data exchange across different institutions.

According to a 2019 Office of the National Coordinator report, around 50% of nonfederal acute care hospitals face electronic data exchange limitations with public health agencies [3]. Of these, 40% struggle with system interface issues that hinder data transmission, while 20% report difficulties because of the lack of a standardized vocabulary between hospitals and public health agencies, further complicating data sharing. Particularly, smaller, rural, and independent hospitals encounter challenges in extracting relevant data from EHRs for reporting purposes. The Office of the National Coordinator report found that 71% of hospitals experience significant challenges in public health reporting, which could limit public health agencies’ ability to monitor and respond to disease outbreaks effectively.

A 2023 report by the Government Accountability Office similarly notes that while electronic health information exchange is widespread, smaller and rural hospitals continue to face obstacles [4]. The report summarized the results of a survey; small hospitals frequently rely on mail or fax, with 47.4% often sending and 54.5% often receiving information through these methods. In contrast, medium or large hospitals use mail or fax less frequently, with 30.3% often sending and 38.5% often receiving data this way. For regional, state, or local health information exchange organizations, 36.7% of small hospitals often send data and 23.2% often receive data, whereas medium or large hospitals are more engaged with these networks, with 56.4% often sending and 40.3% often receiving data. Similarly, for EHR vendor–based networks, 30.2% of small hospitals report sending data and 28.4% report receiving data, while medium or large hospitals show higher use, with 48.4% often sending data and 45.5% often receiving data through these networks. National health information exchange networks also show a gap, as 28.5% of small hospitals often send data and 23.3% often receive data, compared to 43.1% of medium or large hospitals often sending data and 36.5% often receiving data. Limited financial and technical resources, such as insufficient IT staffing, often hinder these hospitals’ ability to sustain consistent electronic exchange [4].

Initiatives like the Trusted Exchange Framework and Common Agreement (TEFCA) aim to create a more unified data exchange approach [5]. TEFCA was established under the 21st Century Cures Act, which was signed into law in December 2016. Specifically, TEFCA aims to improve patient care, reduce health care costs, and enhance health care quality by enabling seamless data exchange among diverse health care entities. Although it is a promising initiative, participation remains voluntary and does not fully resolve these infrastructural challenges.

These findings were supported by a report from the Greater New York Hospital Association (GNYHA) [6], which highlighted the burdens experienced by hospitals during the initial months of the COVID-19 pandemic. The GNYHA report echoed the Office of the National Coordinator’s findings, emphasizing the importance of reviewing data collection frequency and making necessary adjustments to strike a balance between public health requirements and hospitals’ reporting capabilities. Since the onset of the COVID-19 pandemic, the response capacity of the health care workforce has been severely impacted, leading to acute stress reactions and illness among health care professionals [7]. These reactions were especially acute in small hospitals. Health information systems play a vital role in hospitals’ efficient data collection and sharing of data. Such efficiency is valuable at all times, but particularly during a pandemic [8], because accurate and timely data on patient morbidity, mortality, and laboratory findings and institutional bed availability, personal protective equipment supply burn rates, and other concerns (eg, unit staffing and resourcing) are needed to guide decisions for a range of stakeholders involved in the public health response at city, state, and federal levels [8].

### Objectives

The objectives of this paper are (1) to quantify the changing reporting requirements overtime during the pandemic, (2) to characterize the effect of reporting demands on a small independent safety net hospital with an LNH, and (3) to compare the effects of reporting requirements on the SIH and LNH.

## Methods

### Quantifying Reporting Burden

To illustrate the complexity of reporting, we quantified changes to federal reporting requirements over a 14-month period during the COVID-19 pandemic by examining the log of changes and requirements in the Health and Human Services Teletracking, a web-based system for reporting on hospital capacity. We also used interviews and documents to clarify requirements.

### Participants

We interviewed various stakeholders in each institution, including hospital leadership (eg, chief executive officer, chief medical information officer, and chief nursing officer), directors of various hospital departments (eg, infection control, infectious disease, and respiratory therapy), emergency response management team, IT staff, and members of other services.

### Settings and Data Collection

Stakeholders, such as the SIH, LNH, GNYHA, and NYC Department of Health and Mental Hygiene were interviewed using a semistructured interview approach using Zoom (Zoom Video Communications, Inc). Interviews were typically 60 minutes in length. Questions focused on decisions, work activities, tasks associated with each activity, resources used to accomplish those tasks, and institutional goals and challenges to realizing those goals during the pandemic. See Multimedia Appendix 1 for the interview script.

Interviews were transcribed and coded using MAXQDA qualitative analysis software [9] by 5 coders working on different transcripts. Coding was reviewed by an independent coder for consistency and iteratively revised.

### Data Analysis

We used the Systems Engineering Initiative for Patient Safety (SEIPS) model as a theoretical framework and as a basis to analyze the data [10-12]. Grounded in the macroergonomic work system model by Carayon and Smith [13], SEIPS offers a comprehensive approach to understanding the elements of a work system, making it highly relevant to health care environments. The model illustrates how work systems influence care processes, such as care pathways, patient journeys, and workflows, while linking patient outcomes with organizational and employee outcomes. This highlights not only patient safety but also worker safety and quality of working life. The SEIPS model consists of 5 key elements—person, organization, technology and tools, tasks, and environment—enabling its broad applicability [10]. It provides a robust framework for analyzing health care work, particularly under conditions of stress or disruption.

Central to our analysis are the concepts of resilience and adaptive capacity, both integral to the SEIPS framework. Resilience refers to a health care system’s ability to adjust, recover, and continue functioning effectively in the face of disturbances, variability, or adverse events. Adaptive capacity refers to the ability of a system or its individuals to adjust to new circumstances and improve processes. The SEIPS model connects adaptive capacity to how the various work system elements interact to maintain safety and performance in dynamic conditions.

Once interviews were conducted, they were transcribed, cleaned, and coded using a schema based on the SEIPS 2.0 models [10]. The SEIPS model and coding scheme was used to identify the critical aspects of the organization and its associated decision-making, tasks, and experience, which facilitated or challenged the organization’s ability to maintain resilience. The codings schema included 13 top-level codes based on a modified version of the SEIPS model developed by Carayon with additional concept codes specific to this context [11]. The focus of this paper is predominantly on infection control and reporting burden.

### Ethical Considerations

All procedures were approved by the Downstate Medical University Institutional Review Board (1638083-7). Anonymized data was stored on encrypted servers or hard drives in locked offices, and referred to only by a code. No compensation was given to interviewees.

## Results

### Overview

We interviewed 51 people, including 32 at the SIH, 16 at the LNH, and 3 with 2 external agencies, GNYHA and the NYC Department of Health and Mental Hygiene.

### Quantifying Reporting Burden

**Figure 1:**
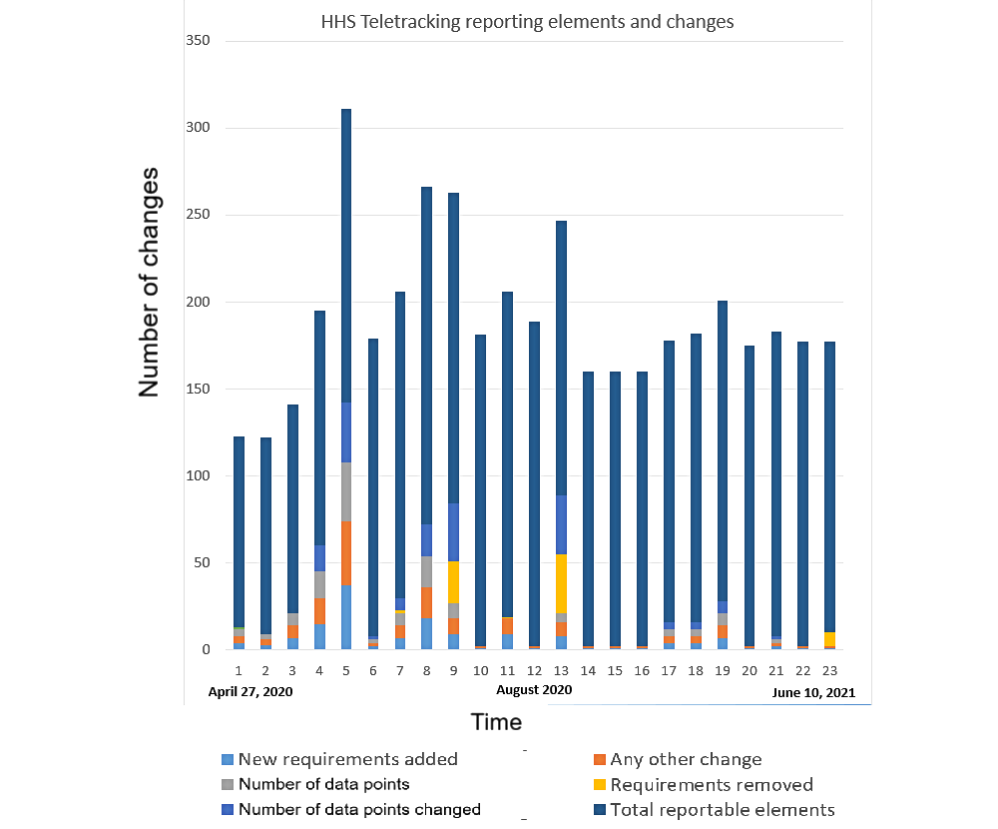
Changes in federal reporting requirements via the Department of Health and Human Services (HHS) Teletracking service.

Reporting clinical and public health data such as numbers of tests, infections, hospitalized patients, and deaths from COVID-19 were mandated by NYC at city, state, and federal levels, via several different agencies and jurisdictions. Table 1 shows the data and agency and provides examples of reporting requirements. The timeline in Figure 1 shows how these have changed over time for the federal Human and Health Services Teletracking system. The graph quantifies burden on reporting staff, showing total changes that reporting staff must handle for each time period. This includes new requirements being added, the total number of data points, and data points which were changed (which also necessitates work even if requirements were removed), as well as total reportable elements. All these together constitute burden. The data were gleaned from the interviews, publicly available documents, and guidance from the emergency response management team that oversaw the process.

The bulk of reporting was to the NYC and New York State departments of health. The schedules varied from several times a day to intermittently or as requested. During the first few months of the COVID-19 pandemic, the reporting requirements increased dramatically. At the SIH, 17 departments were engaged in the reporting process. Nursing and pathology were each involved in 7 reports. The New York State Department of Health reporting was more demanding than the others, and as we discussed, infection control was the most heavily impacted. There were sharp increases and changes across agencies. At the beginning, there were 5 reports which later increased to 29.

Figure 1 includes the total number of data elements, changes in reporting requirements, and other changes. Each of the columns represent a date between April 27, 2020, and June 10, 2021. The columns reflect the 23 dates when a change was made to reporting in that time interval. Of the total 23 changes, 18 changes occurred in 2020, and 6 changes were in July (columns 5-10). The stacked plot shows the total number of data elements to which reporters had to pay attention. On a couple of occasions, data elements were removed, and this was also reflected in the changes. Any change, including removal, could have serious effects on data gathering and processing workflows and personnel. The federal data elements increased sharply at the beginning from 110 to around 170, which coincided with the introduction of the Teletracking system and fluctuated after that.

**Table 1:** Reporting requirements for New York City institutions.

Agency	Data	Submission schedule	SIH^a^ responsible departments
NYCDOH^b^, NYS^c^	Patients with COVID-19 and their bed summary	Daily by 4 PM (later 1 PM)	Nursing, infection control, and risk management
NYCDOH, NYS	Data for COVID-19 pandemic bed increase directive	Onetime request	Emergency management and admitting
NYCDOH, NYS	Fatalities (particularly related to COVID-19)	Within 24 h of death	Pathology, infection control, and nursing
NYCDOH, NYS	PPE^d^, split ventilators, and staffing	One time request	Central supplies, respiratory therapy, and nursing
NYCDOH, NYS	COVID-19 death certification	Within 24 h of death	Pathology and admitting
NYCDOH, NYS	Dialysis needs: staff, supplies, and equipment	Onetime request	Dialysis, central supplies, and nursing
NYCDOH	Pediatric patients with COVID-19	Daily	Nursing, pediatric infectious diseases, admitting, and risk management
NYCDOH	NYCDOH syndromic surveillance ED^e^ data	Daily	IT and emergency department
NYCDOH	Pediatric multisystem inflammatory syndrome	One time request	Nursing, pediatric infectious diseases, admitting, and risk management
NYCDOH	Syndromic surveillance data and patient ID	Daily	IT and emergency department
NYCDOH	Temporary ED data	One time request	Emergency department
NYCDOH	NYC^f^ communicable diseases preparedness	One time request	Emergency management and infection control
HHS^g^	COVID-19 test data from in-house and commercial laboratories	Daily by 5 PM	Pathology and microbiology laboratory
OASAS^h^	COVID-19	One time request	Pathology and microbiology laboratory
GNYHA^i^	Sit Stat daily report	Daily by 11 AM	Emergency management
GNYHA	Inpatient psychiatric census	One time request	Psychiatry, infection control, and nursing
GNYHA	Nursing home EO^j^ impact	2 times/wk	Emergency management
OCME^k^	Hospital morgue census or availability	Daily by 3 PM	Pathology and laboratory
AHA^l^	Hospital laboratory data	Daily	Pathology and microbiology laboratory
HANYS^m^	COVID-19 extraordinary hospital and health system cost	One time request	Emergency management, hospital administration, hospital finance, and clinical services
National Guard	Bed capacity and census	Daily every 12 h	—^n^
CMS^o^	Average cost for specified outpatient drugs	By May 1	Pharmacy, hospital administration, and hospital finance
FEMA^p^	Number of COVID-19 tests and type, antibody test, antibody, and PCR^q^	Daily report due by 5 PM	Pathology and microbiology laboratory

^a^SIH: small independent hospital.

^b^NYCDOH: New York City Department of Health and Mental Hygiene.

^c^NYS: New York State Department of Health.

^d^PPE: personal protective equipment.

^e^ED: emergency department.

^f^NYC: New York City.

^g^HHS: Health and Human Services.

^h^OASAS: Office of Addiction Services and Support.

^i^GNYHA: Greater New York Hospital Association.

^j^EO: emergency office.

^k^OCME: Office of the Chief Medical Examiner.

^l^AHA: American Hospital Association.

^m^HANYS: Health Administration of New York State.

^n^Missing data.

^o^CMS: Center for Medicare and Medicaid Services.

^p^FEMA: Federal Emergency Management Administration.

^q^PCR: polymerase chain reaction.

Definitions changed periodically and created significant stress for those involved in the reporting process. The number of staffed beds versus “available beds” was an example of how definitions were a source of miscommunication. In addition, different agencies used different variables for the same measure regarding bed availability, surges, surge capacity, and so forth. Sometimes, the variables were redefined midstream, necessitating a change in data gathering. It was also problematic that the SIH had 376 total licensed beds but only 198 were staffed. This resulted in discrepancies as it is used as a denominator to estimate available capacity (eg, beds available immediately for an emergency), causing consternation and disputes with agencies.

### Interviews

The interviewees included hospital leadership, medical directors, frontline clinicians, directors of laboratory, nursing, infection control, and other services; their technical staff; those in charge of facilities and logistics; emergency staff; and hospital staff.

Table 2 includes concepts mentioned in the same context (eg, coding segment) as an information report. The state government was the most focally discussed concept in this context (ie, 111 instances). It is the primary recipient of mandatory reporting and posed issues for individuals and institutions responsible for the reporting. Federal agencies were mentioned in 62 instances. Information gathering was seen as a rate-limiting factor in the reporting process because of interoperability and data extraction problems. The interviewees often discussed the arduous nature of the reporting process and how it constituted a burden. Infection control reporting requirements emerged as a central focus. The data gathering involved a collective and often manual process. However, the infection control and infectious disease departments shouldered a disproportionate burden. Particularly, the infection control department had substantial difficulties executing their routine hospital surveillance tasks during the pandemic.

**Table 2:** Concepts co-occurring in the same segment of text (typically a sentence or two) with the code “information report” (n=423).

Variable	Instances, n (%)
Agencies: state	111 (26.2)
Agencies: federal	62 (14.6)
Automated vs manual	46 (10.9)
Information gathering	40 (9.5)
Burden	33 (7.8)
Infection control	29 (6.9)
COVID-19 tests	24 (5.7)
Communication	15 (3.5)
Deaths	15 (3.5)
Patient census	15 (3.5)
Technology tools	13 (3.1)
Agencies: city	10 (2.4)
Work processes	10 (2.4)

### Emergent Themes

#### Differences Between the Two Hospital Systems

There was a stark difference in the impact of reporting requirements on staff at each institution. The LNH had a 50-person analytic team that handled reporting tasks, including data collection and submission. The involvement of clinical staff in reporting was limited to approval and coordination. Reporting was not as much a focal point in LNH interviewees’ responses, even though the interviewers asked about it specifically. By contrast, reporting was a cross-cutting theme in the SIH interviews, with 90% (29/32) of interviewees mentioning it, often in the context of barriers and burdens.

#### Time Constraints, Penalties, and Other Factors Affecting the Task

Typically, new reporting requirements for NY State were first conveyed to institutional contacts at 8 AM with a required deadline of 1 PM the same day. Penalties for missing reporting deadlines posed challenges for the SIH staff, many of whom also faced increased clinical demand during the COVID-19 pandemic which was further complicated by staff shortages. Not only could there be a lack of clarity around new data elements’ definitions, but also, at the SIH, an added reporting requirement could mean nontrivial changes to workflows: conversations with staff, data collection via phone calls or observation, manual extraction from systems without automated exports, tabulation in spreadsheets, and manual entry into reporting forms. Furthermore, the continuous changes made it difficult to develop stable data gathering and workflow processes.

The excerpts in Tables 3 and 4 illustrate the challenges experienced by various personnel involved in regulatory reporting.

As seen in Table 3, the comments from SIH personnel (ie, quote numbers 1-6) highlight the challenges and urgency of meeting reporting requirements and the problems associated with locating and extracting the needed information (ie, quote number 3). Moreover, the burden of reporting may have diminished the resources needed for critical care decisions (ie, quote number 5). The ability to display data in dashboards and use it for predictive modeling was also a major need (ie, quote number 6).

In contrast, the LNH had ample resources from the baseline before the pandemic. However, they also faced challenges associated with the distribution of responsibilities and resources (ie, quote number 7) and experienced the pressures of needing to report in a timely fashion (ie, quote number 8). The dedicated analytics team managed data aggregation and reporting, largely eliminating the burden on other staff. They also had a centralized data warehouse that automatically updated, reducing the need for manual searching and compiling data for reports. Their electronic medical record generated reports that could be accessed by anyone, not just the IT team.

Table 5 shows the distilled data from interviews and documents and compares the reporting process at the SIH and LNH.

There are vast differences in the technical capabilities between the SIH and LNH, typical of the gap between wealthy private hospital networks and small stand-alone hospitals, as seen in Table 5. The manual information gathering processes at the SIH and the fact that the LNH has 50 people involved in data aggregation and analytics, starting from an already highly automated aggregation system, led to profound differences in the reporting workflow.

Table 5 calls attention to several issues pertaining to the processes involved and what might be needed to improve overall. Automation levels, which directly affect burden, are only part of the problem.

**Table 3:** Illustrative quotes.

Quote number	Quotes from the SIH^a^	Codes
1	“For the Department of Health in New York State, they want the report at a set time. If you don't put it in at that time, you get penalized, and it's a huge fine for the hospital. So now we have to meet all the mandates together with meeting your primary responsibilities before COVID, right?” [Infection control leadership, SIH]	Situated resilience: respond or respond to task demands Task: info report Task: burden External environment: state
2	“We have to extract it, so it's not a 100% foolproof process. Sometimes you have to do manual extraction, which is very tedious, and that takes the whole time. So between nine to one-o-clock, that's what we're working on.” [Infection control leadership, SIH]	Task: information gathering, person or domain: nursing Task: burden External environment: state
3	“We have a number of machines that are not interfaced yet with our EMR^b^ for one technical reason or another, and so instead, the technician is responsible for manually entering each result...” [Laboratory administration, SIH]	Tech tools: automated Internal environment: technology
4	“Nobody's analyzing the cases as they come into the hospital. What's their O2 stat in an aggregated way? How sick are they? Are they having fevers? Are they getting discharged? How long has it taken for them to be discharged? I would be really interested in that sort of thing to understand the complexion of the epidemic.” [Infectious disease leadership, SIH]	Structural resilience: capacity to monitor patients Tasks: information gathering Info type: patient census Potential solution
5	“We didn't even know what it meant to be anointed, a COVID-only hospital. Would we get busloads of people? We immediately had to draft scenarios. We have our main hospital and a surgery center a couple of miles away, and we drafted two different models for each site. What if we were a COVID-only hospital at 50% critical care level and then 80% critical care level; what would we need?” [Clinical services, SIH]	Person domain: critical care or ICU^c^ Tasks: predict Adaptation: planned or anticipated Systemic resilience: anticipate demands or capacity misalignments
6	“It’d be also nice to have longitudinal data, of course as well, so taking a look at how things have evolved over the past week over the past month, over the past two months, just to see how those things have changed over time, so I've heard mention of a dashboard. But I haven't seen it implemented yet.” [Laboratory administration, SIH]	Person domain: PPEd supplies Tech tools: potential solution Tech tools: dashboard visualization Structural resilience: monitor or monitor opportunities
7	“[D]uring the beginning I was intubating patients at a rate of one per hour, saving lives. Then I was taken off to save lives by filling out spreadsheets.” [Emergency physician, SIH]	Person domain: emergency medicine Respond: respond to task demands Adaptation: reaction or unanticipated

^a^SIH: small independent hospital.

^b^EMR: electronic medical record.

^c^ICU: intensive care unit.

^d^PPE: personal protective equipment.

**Table 4:** Illustrative quotes.

Quote number	Quotes from the LNH^a^	Codes
8	“[Named person] was one of the people responsible for filling that out early on, and it was one, trying to figure out the definitions, was it accurate, did her... she had to get it quickly approved through the chief operating officer before it would go out centrally, and at the same time, she's trying to build a two hundred-bed field hospital in a week.” [Nursing leadership, LNH]	Tasks: info report Tasks: burden Person domain: executive Person domain: nursing
9	“So there were challenges during that time period that... this was sort of like, ‘Why is this necessary? We're just trying to survive here.’ And yet, it was necessary to understand what was happening in the State of New York, so when you think about it logically, of course, we needed to provide data about what was happening in our hospitals, but at the time when you're choosing between... are you gonna create a bed to try to take care of another patient, or are you gonna slow down enough to present accurate data? It was annoying because it couldn't work.” [Pharmacy leadership, LNH]	External environment: State government Monitor: monitor task demand or capacity misalignment Tasks: info report Tasks: burden

^a^LNH: large network hospital.

**Table 5:** Comparison of the reporting processes at the SIH^a^ and LNH^b^.

	SIH	LNH
HHS^c^ daily report (remdesivir data, monoclonal antibody, COVID-19 positives, pediatrics, and bed capacity)	Rely on department collection of info into Microsoft Excel Small differences in definitions and strict deadlines Remdesivir data from pharmacy Reliance on paper and Microsoft Excel	NSHN^d^ first report March 30, 2020—3 days to prepare, ICU^e^, ventilator counts, 15 nurse team, coordinated by senior vice president or chief operating officer
HERDS^f^, NYS^g^	Daily manual inventory of intake or outflow Need for real-time inventory amplified by crisis PPE^h^: central supply email and Microsoft Excel; phone calls used to reconcile information (eg, sizes and models) Hard to track supplies from orders, expected time of arrival, delivery, and use Historical data kept in spreadsheets No centralized place to share equipment data Coordinated on needs with procurement, central sterile, infection control, disaster management, and facilities management Admissions of patients who tested positive for COVID-19 infection from EMR^i^ in mornings; internal isolation list emailed to leadership	Initial: Manual data entry for each patient; requested extra time as this was impossible during high loads Infection preventionists and regional hospitals submitted manual reports via phone, email, and fax, populated an initial database and Microsoft Excel Used for accuracy tracking to develop automated reports, suspect cases were hard to capture Rapid multiple changes and penalties for not submitting in a few hours State required someone available to answer questions 24/7. Manually intensive Later: Automated via laboratory, centralized reporting from 2 laboratories, saved in Cerner, generated reports 4 times per day.
HERDS: Demographics of patients with COVID-19 (age, employment and essential worker status, how they get to work, and where they were admitted from): deaths. Initiated several months later.	Need for HERDS report to be submitted by 1 PM (working on it from 9 AM to 1 PM). Manual extraction and work with IT to automate or integrate into admission records so that these things are noted by nurses. Lapses where nurses forget to ask these questions. HERDS has to be manually input. Someone else has to attest to it before submission. Deaths by COVID-19: deaths and mortalities via an IT-generated report in Microsoft Excel emailed to attester. COVID-19 discharges: from an IT-generated report emailed to attester. Compare across days with the internal isolation list to ensure that they did not miss any discharges.	HERDS required 2-h turnaround (later 3 h) daily. Analytics dept consisting of clinicians do registry reporting. Both centralized and decentralized—some campuses had capacity, others did not. End of year: 90% HHS reports automated. Medication reporting: by pharmacy, 13 hospitals via email to one person daily, (then weekly, monthly, over) Vitals, patients, inventory, no inventory system, spot-checking, manual decrementing, 2 h per day for pharmacy person. “Paralyzing.” Report via a smartsheet application. Vague definitions initially. [staff] filed, had to get it quickly approved by the chief operating officer, while building 200 bed field hospital in a week. HERDS reporting now done by 20-person team.

^a^SIH: small independent hospital.

^b^LNH: large network hospital.

^c^HHS: Human and Health Services.

^d^NSHN: National Healthcare Safety Network.

^e^ICU: intensive care unit.

^f^HERDS: Health Electronic Response Data System.

^g^NYS: New York State Department of Health.

^h^PPE: personal protective equipment.

^i^EMR: electronic medical record.

#### Ability to Change

Although manual processes are time-consuming and manually coordinated, process changes cannot be easily implemented; unlike the automated data extraction process that takes 10 minutes, there is far less leeway for errors or further changes. Because manual processes require the availability of specific personnel, changes may require additional coordination and sometimes staffing and may not be feasible. Staff retraining may be needed.

#### Institutional Self-Knowledge

Our studies revealed that self-knowledge by the institution is also lacking—our research served to inform staff internally of who was doing what in other areas outside their own, enabling us to pull together an overview and details hitherto unknown by administrators (ie, “organizational situational self-awareness”). Again, this can be a function of lack of IT resources and knowledge of how to use them; such overviews are commonly made in IT process changes where the “as is” and “to be” workflows are explicitly illustrated using convention-based diagramming and planning tools.

#### Effect on Clinical Work

The current processes took clinical staff away from urgent clinical duties, and the effects of this on actual patient outcomes are difficult to quantify, but concerning, particularly for this already underserved population.

It is notable that even common tools, such as shared drives, were not necessarily used (eg, “no central space to share equipment data”). Implementing changes to incorporate digital tools is further hampered by the need to involve IT staff who are overwhelmed by daily duties and emergency needs and unable to consider further process changes even though it would relieve burdens in the long run. Clinical staff made use of external digital tools, such as WhatsApp for coordination as these are freely available, can be set up by clinicians, and meet security requirements. This illustrates what is needed to be useful in future.

However, there were also similarities and common challenges. Neither institution adequately planned for a pandemic of this magnitude. Both had to change rules and procedures and make numerous adaptations. The SIH also had to deal with state and other mandates arising from its status as a COVID-19–only hospital and as part of the state hospital system. This meant that it could not refuse patients with COVID-19 but did not receive extra compensation. There was a significant revenue loss because of canceled surgeries and other procedures. This burden affected all hospitals, but the ones which were resource-limited were more greatly impacted.

## Discussion

### Major Findings and Implications

#### Differences Between the Two Hospital Systems

The COVID-19 pandemic placed a great burden on health care institutions throughout the country. SIHs disproportionately felt the burden. Regulatory reporting is an essential aspect of public health and takes on greater importance during a crisis like the COVID-19 pandemic. Unfortunately, the reporting requirements added to the burden and the great stress these institutions were already experiencing, with a more significant impact on SIHs.

Health IT inequities have been well-documented, and their detrimental impact on health disparities, exacerbated by the COVID-19 pandemic, has been noted [14].

We found that the lack of health IT infrastructure and staffing imposed significant demands on clinical staff and rendered patient care more difficult. We compared the impact on an SIH with a much larger and better-resourced network hospital. Although both endured substantial challenges during the COVID-19 pandemic, the smaller institution disproportionately felt the effect. The larger one had sufficient staffing and the capabilities to support evolving reporting needs, supported by greater automated data extraction capabilities. The findings are consistent with an ONC survey of hospitals conducted before the COVID-19 pandemic, which suggested that many hospitals were ill-prepared [3].

Moreover, the distribution of federal COVID-19 relief funds to hospitals often failed to prioritize the communities most in need [15]. However, it is the institutional inequities, particularly concerning disparate IT infrastructure and automation capabilities, that are deeply concerning and pervasive. While variations between the SIH and the LNH were expected, the differences were more profound and had far-reaching consequences. To establish a truly equitable public health infrastructure that meets the requirements of a modern health care system, adequate resources must be allocated to support the implementation of mandates. This includes funding for training programs and establishing measures to address the disadvantages faced by SIHs. IT has become the backbone of health care, and it is crucial for public health agencies to acknowledge that hospitals with limited IT resources will struggle to fulfill reporting requirements. When feasible, demand should be adjusted or, at the very least, temporal demands should be reduced to accommodate the hospital reporting capacity. Recognizing the disparities in IT capabilities and addressing them is a crucial first step toward building a more equitable health care system. By acknowledging and addressing the challenges faced by resource-limited hospitals, public health agencies can foster a more inclusive and effective reporting framework that supports the needs of all health care providers.

#### Time Constraints, Penalties, and Other Factors Affecting Tasks

##### Punitive Measures Compounding Hospital Stress and Duress

What made reporting requirements more stressful was the punitive measures of some agencies. A report filed a few minutes late could result in a letter sent to the hospital president with the threat of disciplinary action. Different agencies sometimes created contradictory mandates. For example, the state threatened a US $1 million fine for giving vaccines to those outside the proper queue category, while the city threatened fines if all the vaccines were not used by a certain date. Because of the complex and difficult logistical and reporting process, institutional managers at the SIH paused the vaccine distribution for some time. The punitive government rules compounded the stress and burden of manually aggregating data, deciphering sometimes ambiguous requests, confusing data formats, and delivering a report by 1 PM. It also diminished available resources for patient care. For example, some clinical staff in infectious disease spent around 2 hours a day gathering, aggregating, and verifying information. Other clinical personnel at the SIH were also taken away from clinical duties to accommodate reporting. Notably, there was a significant increase nationally in hospital-acquired infections in 2020 compared to 2019 [16].

Health care personnel must be more involved in making the rules and consulted on the impact of rule changes. The agencies’ determination of “need-to-know” needs to be reconciled with the impact of sudden changes to reporting requirements. In addition, the need for strict compliance and enforcement through punitive measures needs to be tempered by the impact of such measures on hospital staff.

##### Government Mandates Often Presume a Level of Existing Technology

The United States cannot expect resource-limited institutions to follow all unfunded mandates. This is most evident in circumstances, such as a pandemic, but it is the state of affairs at all times. Furthermore, it is reasonable to speculate that *health IT inequities contribute to health disparities in patients.* What effect does a lack of automated data collection and aggregation have on the data collected for patient management? How does this affect the equity of quality assurance, needs assessment, and research? This merits further research.

The effect on clinical work is one of the most deeply concerning results, with clinical staff being removed from caring for patients, and the systemic issues contributing to a decline in service as well as new needs, innovations, and workarounds.

### Future Needs and Directions

#### The Need for Regional Data Sharing

A recurrent theme was the need for centralized websites where regional hospitals could list their available supplies and other needs. No means were provided to leverage the data reported to public health agencies to foster cooperation regionally to ensure supplies, staffing, medications, and predictive modeling of surges, which are naturally affected by regional infection rates. The SIH, for example, often experienced a 2- to 3-week lag in COVID-19 pandemic waves compared to neighboring hospitals, and regional data would support efforts to make anticipatory changes. On a related note, it is often difficult for hospitals to repurpose the data generated for reporting purposes to address other hospital needs, such as quality control. Regional data sharing could serve to coalesce data and render it more reusable. In addition, public health agencies need to provide more granular data on COVID-19 cases, deaths, and vaccinations available to allocate resources to mitigate disease spread [17].

#### Need to Gauge Hospital IT Readiness for the Pandemic

Currently, NYC funds both large and small hospitals for infrastructure, and there is competition for these resources, with large networks arguing that they have greater needs. However, it is clear from our study that basic resources are not available; the 2 types of institutions start from very different baselines. Individual nonnetwork SIHs lack the minimum resources in IT infrastructure and personnel to function efficiently; rather, they are reliant on the heroic efforts of staff. To establish whether a hospital or health system has instances of inequity in IT resources, it may be useful to formulate a rubric of questions and a scaling system analogous to the Health Information Management Systems Society 7-Level Health IT Maturity Model [18].

#### Need for Centralization of Data Aggregation Within the Institution

One difference between the SIH and the LNH was that the LNH data collection was centralized, reducing the burden of multiple people doing things manually and increasing the consistency and reliability of the data. Resource-limited institutions could benefit from resources to set up similar centralization, including 1 or 2 personnel dedicated to the reporting task.

#### The Need for Health Care Professionals to Insist on Independent Control and Sharing of Their Data With Commensurate Health IT Development

In the competitive landscape where public reputation, patient experience narratives, and scoring (eg, Press Ganey and Hospital Consumer Assessment of Healthcare Providers and Systems scores) have financial consequences, institutions are wary of sharing data. There is a concern that exposure might put them in a bad light, provide a means of competitive attack, decrease their possible benefits from exclusive data access for research or startup activity, or have other consequences. This was heightened during the COVID-19 pandemic. For example, normal research data sharing was often suspended. There is also a lack of reciprocation in which public health agencies make the submitted data with or without additional analyses available to the hospitals. For much of the pandemic, institutions reported to governments without timely aggregated access to these data not “knowing what they already know.” It may help to set up a health care institution–owned network for data sharing and aggregation outside the government, with appropriate governance, to help institutions know information vital for their operation.

A corollary of this need is the fact that new technologies permit much greater creation and control of functionality by end users. The advent of no-code and low-code tools and “end-user design/programming” in other areas has demonstrated how these can sharply reduce costs and time taken while providing the needed functionality [19]. A further issue is that the control of technology by clinicians on the front lines is likely to be a better fit to the tasks and contexts involved and, if it has a suitable architecture, more easily able to meet the rapidly changing needs found in emergency situations. A greater focus on ensuring such suitable tools are incorporated into existing systems will help ensure that they are available when needed. The SIH was recently cited by Joint Commission on Accreditation of Healthcare Organizations as having one of the best-prepared emergency response frameworks, a critical part of which is the principle that emergency processes are in place and rehearsed as part of normal functions, not suddenly implemented during a new emergency.

New ways of capturing data or streamlining data capture are also needed. For example, instead of having nurses collect numbers of patients with COVID-19 in the ward on paper and then transferring it to Microsoft Excel, apps where such counts could be entered on a phone and automatically put into aggregation and visualization tools available to all on the web would be much more efficient. Voice input can be easier and faster.

Therefore, to “get from here to there” requires careful thought about the steps needed to feasibly integrate digital tools into the institution to aid emergency response.

This may require a 2-pronged simultaneous approach as follows: first, conducting tool selection and acquisition by those with the autonomy to use them on their own (as the clinical services did with WhatsApp), and second, planning steps for which IT staff are involved, with additional resources. Generalizable tools, such as the ability to post on the internet with user-selected granularity of who can view, edit, acquire, or change information, may help meet needs while being sensitive to our findings such as institutional reluctance for sharing data with competitors.

#### Need for a Robust National Data Infrastructure

Despite recent large US investments in health IT, the COVID-19 pandemic revealed longstanding gaps and ongoing challenges in the local, national, and global public health information systems and data infrastructure and a concurrent and underlying data and information crisis and need for mitigation strategies [20,21]. The US public health system is overdue for a real-time, technology-driven surveillance and reporting infrastructure to respond adequately to public health emergencies [22]. Tracking daily hospitalization data at a national level would be a major step forward in quantifying the hospital system’s real-time impact. Many public health agencies and hospitals continue to rely on manual processes to gather, collate, and submit data [23]. In addition to inefficiency, it often results in incomplete and missing information and a compromised ability to predict and map infection surges [23]. It is also well-documented that there was a slow uptake of findings from clinical trials. Dron et al [24] argue that this is the time to advance scientific cooperation and shift the clinical research enterprise toward a data-sharing culture to optimize our response in the service of public health.

### The Importance of Equitable Universal Data Capabilities and the Need for EHR Certification Guidelines

The widespread use of EHRs in our health care system should provide fertile ground for more real-time standardized electronic EHR-based public health reporting. In their review of EHR-based surveillance systems, Aliabadi et al [25] identified 6 challenges to EHR-based surveillance as follows: policy and regulatory, technical factors, managerial factors, standardization, financial factors, and data quality. On the federal level, the EHR certification guidelines, alongside the interoperability framework by TEFCA, serve as both incentives and requirements that could enhance EHRs’ capacity to support near real-time public health reporting. From a technical perspective, the 21st Century Cures Act addresses the critical technical issues of interoperability and data sharing [26]. However, as has occurred with similar initiatives, well-resourced institutions are likely to meet these requirements, while others, such as the institution we studied, may lack funding and struggle to comply. This could lead to further digital disparities with consequences for future emergencies. Establishing these requirements as part of permanent EHR certification guidelines, aligned with TEFCA’s interoperability goals, would help address these issues in the long term.

Despite advances in EHR interoperability, many health care organizations still face significant challenges. These stem from the use of proprietary data formats and varied terminologies across different systems. The absence of a unified data model complicates efforts to normalize and exchange information seamlessly, leading to inefficiencies in reporting and analysis. In addition, the current certification process does not mandate for implementation of the most up-to-date public health–based interoperability standards (eg, Health Level 7 and electronic case reporting [eCR] Fast Healthcare Interoperability Resources [FHIR]).

The US Centers for Disease Control and Prevention has implemented a rule requiring eCR [27], but eCR is not currently mandated. As the regulation stands, institutions have until January 1, 2026, to implement eCR FHIR or demonstrate an alternative working method. There is a recommended FHIR app that can be used to implement this as well as technical and process advisories. However, as has occurred with similar initiatives, the likely outcome is that well-resourced institutions will be able to meet these requirements, while others such as the institution we studied, which do not benefit from funding for tribal health settings and Federally Qualified Health Centers, may struggle to comply. This is likely to lead to further digital disparities with consequences for future emergencies. Our study supports and highlights the great need for financial support for all low-resourced settings to make eCR truly national. Establishing this as part of permanent EHR certification guidelines would also help address the issues in the long term.

### Potential Features and Benefits of Automated Reporting

Automated reporting plans would build on the current eCR rule, including automated extraction, consistent data models, the ability to rapidly specify new data elements and sources (not currently included), rules outside the current triggers that focus on numerical codes, medications and tests, and a complete end-to-end testing in situ, including the personnel and simulated emergent conditions. This would have benefits including (1) rapid response and surveillance of emergent conditions; (2) little need for programmer involvement during crises; (3) complete data from all sites, giving a truly accurate picture of what is happening as it happens; and (4) far more equitable health system development, with the release of personnel currently highly burdened by reporting requirements. Annual influenza season reporting could be used as a practice initiation exercise to refine requirements and test systems.

EHR certification requirements or guidelines are essential to long-term equity and consistency. As long as functionality is optional and unfunded, lower-resourced settings are unlikely to afford compliance, as we see with the current FHIR mandates, which were much delayed in enforcement. Making eCR a certification requirement also puts the onus on the vendor, who has the capacity and funds to implement it. Pandemic emergency response is critical for the nation, and it would be unwise to leave the current “holes” in our defenses, given the potential exponentially rapid spread of some conditions. A guarantee that functionality is in place before emergencies arise would help ensure response feasibility, rather than having the mad scramble that usually takes place.

### Limitations of the Study

The interviews for this study were conducted approximately 6 to 10 months after the initial wave of the COVID-19 pandemic. It is important to acknowledge that memories during this time frame may be prone to inaccuracies or gaps. However, it is fortunate that despite this potential limitation, the interviews yielded substantial convergence of information, which enhances the credibility of our conclusions. It is worth noting that some potential interviewees declined participation because of their reluctance to revisit the traumatic nature of their experiences. This aspect should be taken into account when considering the representativeness of our findings.

While the generalizability of our findings may be somewhat limited because of the data collection being focused on 2 specific settings, it is noteworthy that when we shared our findings with affiliated hospitals, we received feedback indicating that many other institutions encountered similar challenges. This suggests a broader applicability of our findings beyond the settings studied.

It is crucial to continue gathering evidence from various institutions and contexts to gain a comprehensive understanding of the widespread problems faced by health care providers during the COVID-19 pandemic.

### Conclusions

Pandemic-related reporting imposes considerable burdens on all institutions. SIHs experienced a greater burden given that they may have had to rely on manual data collection processes. This may pull clinical staff away from critical clinical duties, affecting care. Greater attention, funding, and development of ITs and training and the setup of dedicated personnel for reporting and analytics may decrease this burden. Using a formal assessment rubric to determine the state of health IT readiness across institutions would assist in comparing institutions for the determination of needs. Greater regional coordination and addressing intangible issues in emergency response are also needed to present a better equipped and more resilient institutional future.

## Appendix

Multimedia Appendix 1 Health IT COVID project: interview questions—activity interview.

## Abbreviations

eCR: electronic case reporting
EHR: electronic health record
FHIR: Fast Healthcare Interoperability Resources
GNYHA: Greater New York Hospital Association
LNH: large network hospital
NYC: New York City
SEIPS: Systems Engineering Initiative for Patient Safety
SIH: small independent hospital
TEFCA: Trusted Exchange Framework and Common Agreement

## Data Availability

The datasets generated and analyzed during this study are not publicly available due to privacy and institutional review board requirements but are available from the corresponding author on reasonable request.
