# Supplementary material for: The Impact of the Burden of COVID-19 Regulatory Reporting in a Small Independent Hospital and a Large Network Hospital: Comparative Mixed Methods Study
Source: Online J Public Health Inform. 2025 Mar 26;17:e63681. doi: 10.2196/63681 (PMC11982767; doi:10.2196/63681)
Supplement: Multimedia Appendix 1 [file ojphi_v17i1e63681_app1.docx]

**Appendix**

**HIT COVID Project – Interview Questions – Activity Interview**

**Introduction**

Thank you for agreeing to participate in this interview. The purpose of this interview is to learn more about the tasks and activities you perform in your position in response to the COVID-19 pandemic. These tasks can be those centered around a piece of health information technology, or a more general task.

Our aim in this interview is to understand the specific sequence of events, participants, goals, and challenges or facilitators involved in the task. We also want to better understand the information you need to perform this task and where you obtain this information, as well as what are the outputs, products, or results of this task.

Please note that this interview will be recorded and transcribed; however, we will never use your name in our analyses or in any publication. Do you have any questions before we begin?

**Please note that this is a semi-structured interview. Although we employ a set of specific questions, we anticipate that the interview will be conversational and free flowing. Feel free to raise any other issues not listed below.**

**Introduction Questions**

1. What is your title?
2. Please briefly describe your role.
3. Could you tell us about your main responsibilities, and the systems you are responsible for?

**Activity/Critical Incident Questions**

1. Within your unit or area, what is a main goal or task related to pandemic response? This can be related to patient care, data coordination, supplies management, or other needs.
2. Why do you complete this task? What is its purpose?
3. When and how often do you complete this task?
4. Where does this task take place and what other departments or areas external to your department are involved in this task?
5. Who is involved in this task? Please reference specific job titles or units if you can. Please include yourself if you are involved.

Probe: Can you describe their overall role/responsibility for this task?

1. Let’s start from the beginning. You are about to start this task, what is the first thing that happens? Please walk me through the task.

Probe: And after this is completed, what happens next?

Probe: Who else is involved in this step?

Probe: What do you do this step? Under what conditions?

Probe: What data or information do you need and how/where do you obtain this data/information?

Probe: What information technology tools or resources do you use to complete this step of the process?

Probe: What are some challenges or difficulties in completing this step of the process?

Probe: What is helpful when completing this step of this process?

1. How do you know the task is completed? What are the final outputs or end results?
2. What parts of this task could be improved upon or made easier? Specifically regarding information systems or technology use?
3. What were your ‘lessons learned’?

Probe: What would you have done differently?

Probe: What went well?

Probe: What are outstanding needs? What would be useful?

1. Anything else you want to state.
